# *rac*-4-Carbamoylpiperidinium *cis*-2-car­boxy­cyclo­hexane-1-carboxyl­ate

**DOI:** 10.1107/S1600536812004710

**Published:** 2012-02-10

**Authors:** Graham Smith, Urs D. Wermuth

**Affiliations:** a Science and Engineering Faculty, Queensland University of Technology, GPO Box 2434, Brisbane, Queensland 4001, Australia

## Abstract

In the title racemic salt, C_6_H_13_N_2_O^+^·C_8_H_11_O_4_^−^, formed from the reaction of *cis*-cyclo­hexane-1,2-dicarb­oxy­lic anhydride with isonipecotamide, the cations are linked into duplex chain substructures through both centrosymmetric cyclic head-to-head ‘amide motif’ hydrogen-bonding associations [graph set *R*_2_^2^(8)] and ‘side-by-side’ *R*_2_^2^(14) associations. The anions are incorporated into the chains through cyclic *R*_4_^3^(10) inter­actions involving amide and piperidinium N—H⋯O_carbox­yl_ hydrogen bonds which, together with inter-anion carb­oxy­lic acid O—H⋯O_carbox­yl_ hydrogen bonds, give a two-dimensional layered structure extending along (011).

## Related literature

For examples of structures of 1:1 Lewis base salts of *cis*-cyclo­hexane-1,2-dicarb­oxy­lic acid, see: Smith & Wermuth (2011*a* ▶,*b* ▶). For examples of isonipecotamide proton-transfer salts, see: Smith & Wermuth (2010 ▶). For graph-set analysis, see: Etter *et al.* (1990 ▶). For hydrogen-bonding motifs, see: Allen *et al.* (1998 ▶).
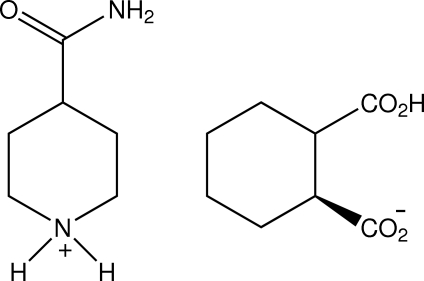


## Experimental

### 

#### Crystal data

C_6_H_13_N_2_O^+^·C_8_H_11_O_4_^−^
*M* *_r_* = 300.35
Monoclinic, 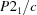

*a* = 19.0097 (14) Å
*b* = 9.0667 (7) Å
*c* = 9.1999 (8) Å
β = 92.022 (7)°
*V* = 1584.7 (2) Å^3^
*Z* = 4
Mo *K*α radiation
μ = 0.10 mm^−1^
*T* = 200 K
0.40 × 0.35 × 0.10 mm

#### Data collection

Oxford Gemini-S CCD area-detector diffractometer
Absorption correction: multi-scan (*CrysAlis PRO*; Oxford Diffraction, 2010 ▶) *T*_min_ = 0.86, *T*_max_ = 0.98
10518 measured reflections
3100 independent reflections
2146 reflections with *I* > 2σ(*I*)
*R*_int_ = 0.057

#### Refinement

*R*[*F* ^2^ > 2σ(*F* ^2^)] = 0.075
*wR*(*F* ^2^) = 0.182
*S* = 1.06
3100 reflections
210 parameters
H atoms treated by a mixture of independent and constrained refinement
Δρ_max_ = 0.43 e Å^−3^
Δρ_min_ = −0.20 e Å^−3^

### 

Data collection: *CrysAlis PRO* (Oxford Diffraction, 2010 ▶); cell refinement: *CrysAlis PRO*; data reduction: *CrysAlis PRO*; program(s) used to solve structure: *SIR92* (Altomare *et al.*, 1994 ▶); program(s) used to refine structure: *SHELXL97* (Sheldrick, 2008 ▶) within *WinGX* (Farrugia, 1999 ▶); molecular graphics: *PLATON* (Spek, 2009 ▶); software used to prepare material for publication: *PLATON*.

## Supplementary Material

Crystal structure: contains datablock(s) global, I. DOI: 10.1107/S1600536812004710/nk2139sup1.cif

Structure factors: contains datablock(s) I. DOI: 10.1107/S1600536812004710/nk2139Isup2.hkl

Supplementary material file. DOI: 10.1107/S1600536812004710/nk2139Isup3.cml

Additional supplementary materials:  crystallographic information; 3D view; checkCIF report

## Figures and Tables

**Table 1:** Hydrogen-bond geometry (Å, °)

*D*—H⋯*A*	*D*—H	H⋯*A*	*D*⋯*A*	*D*—H⋯*A*
N1*A*—H11*A*⋯O41*A*^i^	0.97 (3)	1.95 (3)	2.861 (3)	155 (2)
N1*A*—H12*A*⋯O11	0.99 (4)	1.64 (4)	2.588 (4)	158 (3)
N41*A*—H41*A*⋯O41*A*^ii^	0.86 (3)	2.14 (4)	2.996 (3)	174 (2)
N41*A*—H42*A*⋯O12^iii^	0.77 (3)	2.11 (3)	2.882 (4)	177 (3)
O22—H22⋯O12^iv^	0.93 (5)	1.64 (5)	2.571 (3)	173 (4)

Symmetry codes: (i) 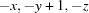; (ii) 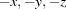; (iii) 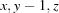; (iv) 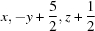.

**Fig. 1.**
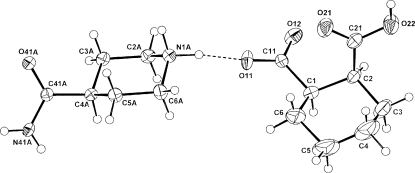
Molecular conformation of the cation and anion in the title compound, with the inter-ion hydrogen bond shown as a dashed line. Displacement ellipsoids are drawn at the 40% probability level.

**Fig. 2.**
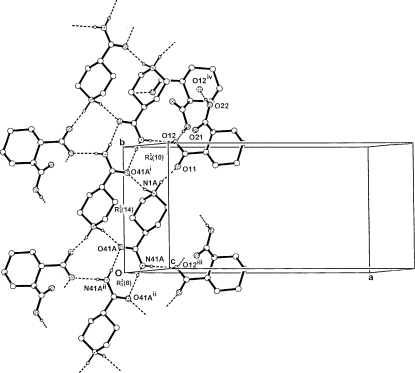
The hydrogen-bonded ribbon substructure in the title salt showing the isonipicotamide cation R22(8) and R22(14) cyclic associations and the R34(10) incorporation of the monoanion. For symmetry codes, see Table 1.

**Fig. 3.**
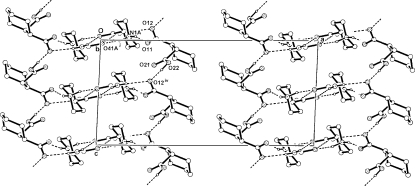
A view of the two-dimensional hydrogen-bonded layered structure looking down the b axial direction, showing the inter-ribbon carboxylic acid···carboxyl hydrogen-bonding extensions down c.

## supplementary crystallographic information

### Comment

*cis*-Cyclohexane-1,2-dicarboxylic anhydride (*cis*-CHDC anhydride) forms racemic 1:1 salts with some Lewis bases and the structures of a few of these have been reported, e.g. with 2-aminopyridine (Smith & Wermuth, 2011*a*) and 4-aminopyridine (Smith & Wermuth, 2011*b*). The 1:1 stoichiometric reaction of *cis*-CHDC anhydride with piperidine-4-carboxamide (isonipecotamide) also gave a racemic salt, the title compound, C_6_H_12_N_2_O^+^.C_8_H_11_O_4_^-^ and the structure is reported here.

In this compound (Fig. 1) the *cis*-configuration of the anion is found as expected, with the cations linked into duplex ribbon substructures through both centrosymmetric cyclic head-to-head hydrogen-bonding associations [the `amide' motif (Allen *et al.*, 1998)] [graph set *R*_2_^2^(8) (Etter *et al.*, 1990)] and `side-by-side' *R*_2_^2^(14) associations (Table 1, Fig. 2). Both of these associations have been found in the structures of Lewis base salts of isonipecotamide (Smith & Wermuth, 2010). In the present structure, the monoanions are incorporated into the ribbons through cyclic *R*^3^_4_(10) amide and piperidinium N—H···O_carboxyl_ associations and together with inter-anion carboxylic acid O—H···O_carboxyl_ hydrogen bonds down *c* (Fig. 3), give a two-dimensional layered structure extending along (011).

### Experimental

The title compound was synthesized by heating together under reflux for 15 min, 1 mmol quantities of cyclohexane-1,2-dicarboxylic anhydride and piperidine-4-carboxamide (isonipecotamide) in 50 ml of methanol. After volume reduction to 30 ml, the hot-filtered solution was allowed evaporate to dryness at room temperature, giving a white amorphous powder. Minor colourless crystal plates were obtained in the residual viscous residue after evaporation of a solution of the compound in 80% propane-2-ol–water.

### Refinement

H atoms potentially involved in hydrogen-bonding associations were located in a difference Fourier analysis and their positional and isotropic displacement parameters were refined. Other H atoms were included in the refinement at calculated positions [C—H = 0.97–0.98 Å] with *U*_iso_(H) = 1.2*U*_eq_(C), using a riding-model approximation.

### Crystal data

**Table:**

C_6_H_13_N_2_O^+^·C_8_H_11_O_4_^−^	*F*(000) = 644
*M**_r_* = 300.35	*D*_x_ = 1.255 Mg m^−^^3^
Monoclinic, *P*2_1_/*c*	Mo *K*α radiation, λ = 0.71073 Å
Hall symbol: -P 2ybc	Cell parameters from 3793 reflections
*a* = 19.0097 (14) Å	θ = 3.2–28.9°
*b* = 9.0667 (7) Å	µ = 0.10 mm^−^^1^
*c* = 9.1999 (8) Å	*T* = 200 K
β = 92.022 (7)°	Plate, colourless
*V* = 1584.7 (2) Å^3^	0.40 × 0.35 × 0.10 mm
*Z* = 4	

### Data collection

**Table:**

Oxford Gemini-S CCD area-detector diffractometer	3100 independent reflections
Radiation source: Enhance (Mo) X-ray source	2146 reflections with *I* > 2σ(*I*)
Graphite monochromator	*R*_int_ = 0.057
Detector resolution: 16.077 pixels mm^-1^	θ_max_ = 26.0°, θ_min_ = 3.2°
ω scans	*h* = −23→22
Absorption correction: multi-scan (*CrysAlis PRO*; Oxford Diffraction, 2010)	*k* = −11→11
*T*_min_ = 0.86, *T*_max_ = 0.98	*l* = −11→11
10518 measured reflections	

### Refinement

**Table:**

Refinement on *F*^2^	Primary atom site location: structure-invariant direct methods
Least-squares matrix: full	Secondary atom site location: difference Fourier map
*R*[*F*^2^ > 2σ(*F*^2^)] = 0.075	Hydrogen site location: inferred from neighbouring sites
*wR*(*F*^2^) = 0.182	H atoms treated by a mixture of independent and constrained refinement
*S* = 1.06	*w* = 1/[σ^2^(*F*_o_^2^) + (0.0845*P*)^2^ + 0.9949*P*] where *P* = (*F*_o_^2^ + 2*F*_c_^2^)/3
3100 reflections	(Δ/σ)_max_ = 0.002
210 parameters	Δρ_max_ = 0.43 e Å^−^^3^
0 restraints	Δρ_min_ = −0.20 e Å^−^^3^

### Special details

**Table:**

Geometry. Bond distances, angles *etc*. have been calculated using the rounded fractional coordinates. All su's are estimated from the variances of the (full) variance-covariance matrix. The cell e.s.d.'s are taken into account in the estimation of distances, angles and torsion angles
Refinement. Refinement of *F*^2^ against ALL reflections. The weighted *R*-factor *wR* and goodness of fit *S* are based on *F*^2^, conventional *R*-factors *R* are based on *F*, with *F* set to zero for negative *F*^2^. The threshold expression of *F*^2^ > σ(*F*^2^) is used only for calculating *R*-factors(gt) *etc*. and is not relevant to the choice of reflections for refinement. *R*-factors based on *F*^2^ are statistically about twice as large as those based on *F*, and *R*-factors based on ALL data will be even larger.

### Fractional atomic coordinates and isotropic or equivalent isotropic displacement parameters (Å^2^)

**Table:**

	*x*	*y*	*z*	*U*_iso_*/*U*_eq_	
O11	0.21833 (13)	0.8455 (3)	0.0243 (3)	0.0522 (9)	
O12	0.23582 (11)	1.0432 (3)	−0.1098 (2)	0.0415 (8)	
O21	0.25201 (12)	1.1290 (3)	0.2369 (3)	0.0487 (9)	
O22	0.31747 (13)	1.3299 (3)	0.2117 (3)	0.0485 (9)	
C1	0.33119 (16)	0.9592 (3)	0.0442 (4)	0.0362 (10)	
C2	0.35236 (15)	1.1183 (4)	0.0836 (4)	0.0337 (10)	
C3	0.42799 (19)	1.1251 (5)	0.1462 (5)	0.0634 (16)	
C4	0.4392 (2)	1.0232 (6)	0.2741 (6)	0.086 (2)	
C5	0.4211 (2)	0.8668 (6)	0.2325 (6)	0.085 (2)	
C6	0.3456 (2)	0.8519 (4)	0.1711 (5)	0.0586 (14)	
C11	0.25621 (15)	0.9481 (3)	−0.0177 (3)	0.0306 (9)	
C21	0.30223 (16)	1.1903 (4)	0.1864 (3)	0.0337 (10)	
O41A	−0.00706 (10)	0.2052 (2)	−0.0339 (2)	0.0298 (7)	
N1A	0.13089 (14)	0.6330 (3)	−0.0339 (3)	0.0293 (8)	
N41A	0.08655 (16)	0.0589 (3)	−0.0669 (3)	0.0286 (8)	
C2A	0.11125 (16)	0.5811 (3)	−0.1821 (3)	0.0290 (9)	
C3A	0.06735 (15)	0.4418 (3)	−0.1727 (3)	0.0257 (9)	
C4A	0.10386 (14)	0.3218 (3)	−0.0845 (3)	0.0251 (8)	
C5A	0.12773 (16)	0.3800 (3)	0.0645 (3)	0.0292 (9)	
C6A	0.17115 (17)	0.5201 (3)	0.0524 (3)	0.0325 (10)	
C41A	0.05649 (14)	0.1901 (3)	−0.0612 (3)	0.0236 (8)	
H1	0.36180	0.92850	−0.03370	0.0430*	
H2	0.35070	1.17570	−0.00670	0.0400*	
H22	0.288 (3)	1.369 (5)	0.281 (5)	0.082 (15)*	
H31	0.46020	1.09850	0.07100	0.0760*	
H32	0.43860	1.22540	0.17640	0.0760*	
H41	0.41000	1.05460	0.35270	0.1030*	
H42	0.48800	1.02810	0.30850	0.1030*	
H51	0.42710	0.80410	0.31750	0.1020*	
H52	0.45340	0.83290	0.16040	0.1020*	
H61	0.31300	0.87170	0.24740	0.0700*	
H62	0.33780	0.75160	0.13760	0.0700*	
H4A	0.14530	0.28900	−0.13600	0.0300*	
H11A	0.0869 (17)	0.663 (3)	0.007 (3)	0.027 (8)*	
H12A	0.161 (2)	0.722 (4)	−0.035 (4)	0.061 (11)*	
H21A	0.15340	0.56100	−0.23520	0.0350*	
H22A	0.08450	0.65690	−0.23390	0.0350*	
H31A	0.02300	0.46550	−0.12910	0.0310*	
H32A	0.05690	0.40530	−0.27020	0.0310*	
H41A	0.0623 (18)	−0.017 (4)	−0.045 (3)	0.037 (10)*	
H42A	0.1263 (17)	0.054 (3)	−0.082 (3)	0.017 (8)*	
H51A	0.08680	0.40010	0.12130	0.0350*	
H52A	0.15560	0.30510	0.11510	0.0350*	
H61A	0.18260	0.55830	0.14880	0.0390*	
H62A	0.21480	0.49810	0.00550	0.0390*	

### Atomic displacement parameters (Å^2^)

**Table:**

	*U*^11^	*U*^22^	*U*^33^	*U*^12^	*U*^13^	*U*^23^
O11	0.0398 (14)	0.0352 (14)	0.0811 (19)	−0.0182 (11)	−0.0039 (13)	0.0027 (13)
O12	0.0305 (12)	0.0545 (16)	0.0393 (13)	−0.0007 (11)	−0.0015 (10)	0.0068 (11)
O21	0.0385 (14)	0.0496 (15)	0.0590 (16)	−0.0112 (11)	0.0170 (12)	−0.0067 (12)
O22	0.0473 (15)	0.0479 (16)	0.0512 (15)	−0.0127 (12)	0.0156 (12)	−0.0159 (12)
C1	0.0240 (16)	0.0363 (19)	0.0483 (19)	0.0031 (14)	0.0016 (14)	−0.0011 (15)
C2	0.0211 (15)	0.0402 (19)	0.0398 (18)	−0.0042 (13)	0.0006 (13)	−0.0027 (14)
C3	0.028 (2)	0.074 (3)	0.088 (3)	−0.0069 (19)	−0.001 (2)	−0.025 (2)
C4	0.043 (3)	0.111 (5)	0.102 (4)	0.009 (3)	−0.035 (3)	−0.007 (3)
C5	0.053 (3)	0.097 (4)	0.103 (4)	0.028 (3)	−0.022 (3)	0.026 (3)
C6	0.046 (2)	0.047 (2)	0.082 (3)	0.0090 (18)	−0.008 (2)	0.020 (2)
C11	0.0273 (16)	0.0242 (16)	0.0403 (17)	−0.0002 (13)	−0.0005 (13)	−0.0073 (14)
C21	0.0277 (17)	0.0436 (19)	0.0294 (16)	−0.0014 (15)	−0.0036 (13)	−0.0003 (14)
O41A	0.0228 (11)	0.0265 (11)	0.0401 (12)	−0.0011 (9)	0.0010 (9)	0.0013 (9)
N1A	0.0274 (15)	0.0253 (14)	0.0351 (14)	0.0002 (12)	−0.0004 (11)	−0.0009 (11)
N41A	0.0188 (14)	0.0291 (15)	0.0381 (15)	−0.0049 (12)	0.0026 (11)	0.0020 (11)
C2A	0.0288 (16)	0.0317 (16)	0.0264 (15)	0.0014 (13)	−0.0010 (12)	0.0075 (13)
C3A	0.0257 (15)	0.0310 (16)	0.0200 (14)	−0.0011 (13)	−0.0046 (11)	0.0013 (12)
C4A	0.0217 (14)	0.0258 (15)	0.0278 (15)	0.0000 (12)	−0.0006 (12)	0.0001 (12)
C5A	0.0326 (17)	0.0284 (16)	0.0259 (15)	0.0002 (13)	−0.0092 (12)	0.0037 (12)
C6A	0.0378 (18)	0.0270 (16)	0.0318 (16)	−0.0027 (14)	−0.0123 (13)	0.0051 (13)
C41A	0.0240 (15)	0.0253 (15)	0.0211 (14)	−0.0014 (12)	−0.0063 (11)	0.0014 (12)

### Geometric parameters (Å, º)

**Table:**

O11—C11	1.246 (4)	C3—H31	0.9700
O12—C11	1.260 (4)	C3—H32	0.9700
O21—C21	1.211 (4)	C4—H42	0.9700
O22—C21	1.317 (4)	C4—H41	0.9700
O22—H22	0.93 (5)	C5—H52	0.9700
O41A—C41A	1.250 (3)	C5—H51	0.9700
N1A—C6A	1.490 (4)	C6—H62	0.9700
N1A—C2A	1.478 (4)	C6—H61	0.9700
N41A—C41A	1.322 (4)	C2A—C3A	1.518 (4)
N1A—H12A	0.99 (4)	C3A—C4A	1.511 (4)
N1A—H11A	0.97 (3)	C4A—C41A	1.515 (4)
N41A—H41A	0.86 (3)	C4A—C5A	1.523 (4)
N41A—H42A	0.77 (3)	C5A—C6A	1.521 (4)
C1—C2	1.538 (5)	C2A—H21A	0.9700
C1—C6	1.537 (5)	C2A—H22A	0.9700
C1—C11	1.520 (4)	C3A—H31A	0.9700
C2—C3	1.531 (5)	C3A—H32A	0.9700
C2—C21	1.514 (5)	C4A—H4A	0.9800
C3—C4	1.505 (7)	C5A—H51A	0.9700
C4—C5	1.506 (8)	C5A—H52A	0.9700
C5—C6	1.530 (6)	C6A—H61A	0.9700
C1—H1	0.9800	C6A—H62A	0.9700
C2—H2	0.9800		
			
C21—O22—H22	111 (3)	C4—C5—H51	109.00
C2A—N1A—C6A	112.4 (2)	C4—C5—H52	109.00
C6A—N1A—H11A	114.9 (16)	H51—C5—H52	108.00
H11A—N1A—H12A	106 (3)	C6—C5—H52	109.00
C6A—N1A—H12A	106 (2)	C1—C6—H62	109.00
C2A—N1A—H12A	112 (2)	C1—C6—H61	109.00
C2A—N1A—H11A	104.7 (17)	C5—C6—H62	109.00
H41A—N41A—H42A	122 (3)	H61—C6—H62	108.00
C41A—N41A—H42A	119 (2)	C5—C6—H61	109.00
C41A—N41A—H41A	119 (2)	N1A—C2A—C3A	109.5 (2)
C6—C1—C11	112.5 (3)	C2A—C3A—C4A	112.7 (2)
C2—C1—C6	112.1 (3)	C3A—C4A—C41A	112.3 (2)
C2—C1—C11	112.6 (2)	C5A—C4A—C41A	107.8 (2)
C1—C2—C21	112.7 (3)	C3A—C4A—C5A	110.5 (2)
C3—C2—C21	110.6 (3)	C4A—C5A—C6A	111.6 (2)
C1—C2—C3	111.2 (3)	N1A—C6A—C5A	110.1 (2)
C2—C3—C4	112.0 (3)	O41A—C41A—C4A	121.7 (2)
C3—C4—C5	110.8 (4)	N41A—C41A—C4A	116.3 (2)
C4—C5—C6	112.3 (4)	O41A—C41A—N41A	121.9 (3)
C1—C6—C5	111.4 (3)	N1A—C2A—H21A	110.00
O11—C11—O12	123.5 (3)	N1A—C2A—H22A	110.00
O11—C11—C1	118.6 (3)	C3A—C2A—H21A	110.00
O12—C11—C1	118.0 (3)	C3A—C2A—H22A	110.00
O21—C21—C2	124.3 (3)	H21A—C2A—H22A	108.00
O22—C21—C2	112.7 (3)	C2A—C3A—H31A	109.00
O21—C21—O22	123.0 (3)	C2A—C3A—H32A	109.00
C6—C1—H1	106.00	C4A—C3A—H31A	109.00
C2—C1—H1	106.00	C4A—C3A—H32A	109.00
C11—C1—H1	106.00	H31A—C3A—H32A	108.00
C3—C2—H2	107.00	C3A—C4A—H4A	109.00
C1—C2—H2	107.00	C5A—C4A—H4A	109.00
C21—C2—H2	107.00	C41A—C4A—H4A	109.00
C4—C3—H31	109.00	C4A—C5A—H51A	109.00
C2—C3—H31	109.00	C4A—C5A—H52A	109.00
H31—C3—H32	108.00	C6A—C5A—H51A	109.00
C4—C3—H32	109.00	C6A—C5A—H52A	109.00
C2—C3—H32	109.00	H51A—C5A—H52A	108.00
C5—C4—H42	109.00	N1A—C6A—H61A	110.00
C3—C4—H42	110.00	N1A—C6A—H62A	110.00
H41—C4—H42	108.00	C5A—C6A—H61A	110.00
C5—C4—H41	110.00	C5A—C6A—H62A	110.00
C3—C4—H41	109.00	H61A—C6A—H62A	108.00
C6—C5—H51	109.00		
			
C2A—N1A—C6A—C5A	−58.9 (3)	C3—C2—C21—O22	59.5 (4)
C6A—N1A—C2A—C3A	58.5 (3)	C1—C2—C21—O21	2.3 (5)
C11—C1—C2—C3	179.7 (3)	C2—C3—C4—C5	57.2 (5)
C11—C1—C2—C21	55.0 (4)	C3—C4—C5—C6	−56.7 (5)
C6—C1—C2—C21	−73.1 (3)	C4—C5—C6—C1	53.8 (5)
C11—C1—C6—C5	−179.3 (3)	N1A—C2A—C3A—C4A	−55.6 (3)
C2—C1—C11—O11	−137.0 (3)	C2A—C3A—C4A—C5A	53.0 (3)
C2—C1—C11—O12	43.4 (4)	C2A—C3A—C4A—C41A	173.4 (2)
C6—C1—C11—O11	−9.1 (4)	C41A—C4A—C5A—C6A	−175.6 (2)
C6—C1—C11—O12	171.3 (3)	C3A—C4A—C41A—O41A	−40.5 (4)
C2—C1—C6—C5	−51.1 (4)	C3A—C4A—C41A—N41A	141.6 (3)
C6—C1—C2—C3	51.7 (4)	C5A—C4A—C41A—O41A	81.5 (3)
C1—C2—C3—C4	−54.8 (5)	C5A—C4A—C41A—N41A	−96.5 (3)
C21—C2—C3—C4	71.2 (4)	C3A—C4A—C5A—C6A	−52.5 (3)
C1—C2—C21—O22	−175.4 (3)	C4A—C5A—C6A—N1A	55.2 (3)
C3—C2—C21—O21	−122.8 (4)		

### Hydrogen-bond geometry (Å, º)

**Table:**

*D*—H···*A*	*D*—H	H···*A*	*D*···*A*	*D*—H···*A*
N1*A*—H11*A*···O41*A*^i^	0.97 (3)	1.95 (3)	2.861 (3)	155 (2)
N1*A*—H12*A*···O11	0.99 (4)	1.64 (4)	2.588 (4)	158 (3)
N41*A*—H41*A*···O41*A*^ii^	0.86 (3)	2.14 (4)	2.996 (3)	174 (2)
N41*A*—H42*A*···O12^iii^	0.77 (3)	2.11 (3)	2.882 (4)	177 (3)
O22—H22···O12^iv^	0.93 (5)	1.64 (5)	2.571 (3)	173 (4)
C4*A*—H4*A*···O21^v^	0.98	2.49	3.340 (4)	145
C2*A*—H21*A*···O21^v^	0.97	2.57	3.389 (4)	143
C2*A*—H22*A*···O41*A*^vi^	0.97	2.59	3.413 (3)	143
C3—H32···O22	0.97	2.52	2.884 (5)	102
C6*A*—H61*A*···O12^vii^	0.97	2.58	3.351 (3)	137

Symmetry codes: (i) −*x*, −*y*+1, −*z*; (ii) −*x*, −*y*, −*z*; (iii) *x*, *y*−1, *z*; (iv) *x*, −*y*+5/2, *z*+1/2; (v) *x*, −*y*+3/2, *z*−1/2; (vi) −*x*, *y*+1/2, −*z*−1/2; (vii) *x*, −*y*+3/2, *z*+1/2.
